# Control, struggle, and emergent masculinities: a qualitative study of men’s care-seeking determinants for chronic cough and tuberculosis symptoms in Blantyre, Malawi

**DOI:** 10.1186/1471-2458-14-1053

**Published:** 2014-10-09

**Authors:** Jeremiah Chikovore, Graham Hart, Moses Kumwenda, Geoffrey A Chipungu, Nicola Desmond, Liz Corbett

**Affiliations:** HIV/AIDS, Sexually Transmitted Infections & TB, Human Sciences Research Council, 750 Mary Thipe Rd, Cato Manor, Durban 4001 South Africa; School of Life & Medical Sciences, University College London, Maple House 1A, First Floor, 149 Tottenham Court Road, London, W1T 7JA UK; Helse Nord TB Initiative, College of Medicine, Private Bag 360, Chichiri, Blantyre Malawi; Malawi Liverpool Wellcome Research Programme, P.O Box 30096, Chichiri, Blantyre 3 Malawi; Liverpool School of Tropical Medicine, Pembroke Place, Liverpool, L3 5QA UK; London School of Hygiene and Tropical Medicine, Keppel Street, London, WC1E 7HT UK

**Keywords:** Malawi, Africa, Masculinity, Tuberculosis, Healthcare-seeking, Gender, Control, Qualitative, Emergent masculinities

## Abstract

**Background:**

Men’s healthcare-seeking delay results in higher mortality while on HIV or tuberculosis (TB) treatment, and implies contribution to ongoing community-level TB transmission before initiating treatment. We investigated masculinity’s role in healthcare-seeking delay for men with TB-suggestive symptoms, with a view to developing potential interventions for men.

**Methods:**

Data were collected during March 2011- March 2012 in three high-density suburbs in urban Blantyre. Ten focus group discussions were carried out of which eight (mixed sex = two; female only = three; male only = three) were with 74 ordinary community members, and two (both mixed sex) were with 20 health workers. Individual interviews were done with 20 TB patients (female =14) and 20 un-investigated chronic coughers (female = eight), and a three-day workshop was held with 27 health stakeholder representatives.

**Results:**

An expectation to provide for and lead their families, and to control various aspects of their lives while facing limited employment opportunities and small incomes leaves men feeling inadequate, devoid of control, and anxious about being marginalised as men. Men were fearful about being looked at as less than men, and about their wives engaging in extramarital sex without ability to detect or monitor them. Control was a key defining feature of adequate manhood, and efforts to achieve it also led men into side-lining their health. Articulate and consistent concepts of men’s bodily strength or appropriate illness responses were absent from the accounts.

**Conclusions:**

Facilitating men to seek care early is an urgent public health imperative, given the contexts of high HIV/AIDS prevalence but increasingly available treatment, and the role of care-seeking delay in TB transmission. Men’s struggles trying to achieve ideal images seem to influence their engagement with their health. Ambiguous views regarding some key masculinity representations and the embrace of less harmful masculinities raise questions about some common assumptions that guide work with men. Apparent ‘emergent masculinities’ might be a useful platform from which to support the transformation of harmful masculinity. Finally, the complex manifestations of masculinity indicate the need for interventions targeting men in health and TB control to assume supportive, multidimensional and long-term outlooks.

## Background

Tuberculosis (TB) is a leading cause of adult morbidity and mortality globally. In 2012, TB was responsible for 8.6 million cases, and 1.3 million deaths of which 0.32 million were in people living with Human immunodeficiency virus (HIV) [1]. Its recent resurgence in Africa has been attributed largely to a combination of the HIV epidemic, weak health systems, rapid urbanization, and poor living conditions in fast-growing cities [2].

Men appear substantially less well served than women by health services, with longer delays in seeking healthcare [3–5], gender disparities in life expectancy increasing, and little impact on adult mortality in men during a decade of substantial global investment in health services. Male gender is a risk factor for late HIV diagnosis and for death either on antiretroviral therapy [6, 7] or TB treatment [1, 8, 9]. Men’s delay in seeking healthcare also has important implications for TB disease control. National surveys consistently show much higher burden of undiagnosed infectious TB in men than women, implying men are the major source of TB transmission events [10–12]. Studies that have examined gender aspects of TB, however, equate gender with the female sex, emphasize women’s perspectives, and frequently suggest that women are more vulnerable than men [13–18]. Systematic approaches that engage men in TB prevention and care are needed. To be successful, these approaches should address the social, cultural, political, and economic processes that generate, reproduce, and sustain masculine identities, roles, and behaviours.

This paper employs a framework that combines different approaches in men’s studies to examine masculinity’s role in healthcare-seeking. According to Connell, “Masculinities concern the position of men in a gender order. They can be defined as the patterns of practice by which people (both men and women, though predominantly men) engage that position” [19]. One of the approaches used here – the ‘critical men’s studies’– holds that gender is socially constructed and gender power relations complex, fluid, and contradictory; there is nevertheless need to pay attention to asymmetrical gender relations [20]. The other stresses how men are facing ‘a crisis’, in part through women’s efforts and successes in rebalancing power inequity [21]. In line with Connell [22], the paper acknowledges the ‘crisis in masculinity’ and sees it as also arising from the heterogeneous and contradictory configurations of gender. In addition, paying attention to gender interactional dynamics [23] recognises the role of women and femininity in shaping masculinity, and of men and masculinity in affecting the health of women and of society at large. This gender relational axis is, in turn, located within a wider social, political, historical and economic context [20, 23].

The paper focuses on the notion of masculine control as it emerged within a qualitative research process. As a central aspect of masculinity, control features more extensively in the global-wide literature compared to studies from Africa. Classical discussions of hegemonic masculinity relate adverse health consequences for men (and women) to the high social value placed on power and privilege. The resulting drive to exercise control through, for example, competitiveness, aggression, and physical strength is seen as carrying direct health consequences for men [20, 24]. Similarly, men’s pressures arising from failure to meet dominant expectations trigger a crisis that drives risk-taking and poor health behaviour [25, 26].

Studies from Africa mainly deal with masculine control in the context of gender-based violence and inequity, and HIV/AIDS sexual risk behaviour [27–33]. More recently, however, studies have begun to highlight men’s experience of vulnerability. This is in view of psychosocial challenges related to HIV/AIDS, and the pressures placed on men most of whom are already poor and marginalized [26, 28, 34–36]. Given the evidence that male gender contributes to poor TB control outcomes, and that rapid and drastic socio-economic and structural changes including in labour markets and legislation/policy are reshaping gender relations within families and the community [25, 26, 34], we explored they ways contemporary notions of masculinity influence TB-related healthcare-seeking in an urban slum setting. Ultimately, our aim was to contribute to the development of candidate interventions targeting men in TB control.

## Methods

### Setting

Malawi is low-income and agriculture-dependent, with a population of about 15 million, two-thirds of whom lived below the poverty datum line in 2010 [37]. It has one of the fastest rates of urbanisation globally [38]. The projected population for Blantyre, the study city, for 2013 was 816,001 [39], and 70% live in unplanned settlements [40]. The study was carried out in three high density locales within these settlements. The population density for Blantyre city is 3,417/m2 compared to 203/m2 in rural parts of Blantyre district [39]. In 2013, the adult national HIV prevalence was 10.3% [41]. National TB incidence is estimated at 163 per 100,000 population, and 78% of cases are diagnosed within a year against the global target of 70%. The case notification rate for Blantyre city has been estimated at 458 per 100,000 in 2013 (Dr EL Corbett, personal communications). At 85%, the TB treatment success rate matches the global target [1]. In 2011, coverage of people in need of antiretroviral therapy was estimated at 76% [42].

### Design, sampling and data collection

This pre-intervention exploratory study was aligned to a larger cross-sectional household survey. The study was carried out in conformity with the RATS guidelines for qualitative research [43]. Data were collected over 12 months from March 2011 using an exploratory approach informed largely by grounded theory [44] in view of limited understandings around the study topic. Prolonged cough – a well-established symptom of pulmonary TB – was the main entry point having considered that few people within the general community could provide TB-specific information without extensive guiding. Moreover, as in other studies [45], the chronic cough-TB link justified using recognition and response to chronic cough as a proxy for TB recognition and response. Different dimensions of the study topic, including beliefs, normative values and personal experiences were explored through triangulating methods and sources, and purposefully choosing and varying the sample by gender [46, 47] (Table 1). A reflexive and iterative process helped enrich tools and build on emerging concepts during fieldwork.

**Table 1:** **Data sources, sample size and recruitment of participants**

Technique	Participant category	N (total participants)	Gender and age	Mode of recruitment
FGD	HCW	2 (20)	mixed sex (3 men and 7 women per group); age 23 – 58)	At PCCs; chosen to include wide range of providers (Nurse midwife technicians, nurses, Health Surveillance Assistants, TB officers, Clinical Officers)
	Community members	8 (74)	3 – men only	Through approaching households
			3 – women only	
			2 – mixed (11 women, 9 men)	
IDI	Newly diagnosed TB patients	20	14 – women	Through registers held at PCCs
			6 – men	
	Chronic coughers	20	8 – women	Through household survey inquiring about cough
			12 – men	
Participatory	Health stakeholders		14 – women	
Workshop			13 – men	

Ten focus group discussions (FGD) were carried out of which eight (two mixed sex, and three each for men and women separately) were with 74 ordinary community members; and two (both mixed sex) were with 20 health workers. In addition, individual interviews (IDI) were done with 20 recently diagnosed TB patients (14 women, six men) and 20 chronic coughers who reportedly had not sought formal care (eight women, 12 men). We also held a three-day participatory workshop with 27 health stakeholder representatives (comprising health service providers and managers, traditional and religious leaders, and ordinary members of the community) to feed back the findings and begin the process of developing potential interventions (the workshop will be reported on in detail separately). Minimum sample sizes were determined taking into account the numbers considered necessary to explore different dimensions of phenomena in qualitative research [48]. Ongoing reflection and informal analysis during data collection also led to a determination, at the end of the first round of data collection, that a fairly complete picture had emerged around the topic, and little new information might be generated from further fieldwork.

Chronic coughers were identified through the survey, which inquired about the presence of chronic cough (defined as cough lasting two weeks or more) within households and noted Global Positioning System (GPS) coordinates of applicable households. Qualitative data collectors then followed up using the GPS coordinates to recruit eligible participants. Newly diagnosed TB patients were identified through TB registers at primary care centres (PCCs). For both groups, their specific experiences of illness symptoms and healthcare-seeking were explored individually. We considered that personal and individual experiences are better explored in private [49]; moreover, interviewing chronic coughers and TB patients in groups would be inappropriate in view of the stigma of and the link commonly made among cough, TB and HIV [50–53].

Community FGD participants were identified by directly approaching households within the study community and recruiting the first adult member to consent to participating in the study, while health workers were recruited at PCCs and selected to cover a broad range of frontline primary care workers. Community FGDs explored normative views of gender roles, and general perspectives of TB, chronic cough, and healthcare-seeking. Health worker FGDs explored their perspectives of how the healthcare delivery system interacted with patients, partly based on what other participants had described in IDIs and community FGDs. In other words, we theoretically pursued and sought to verify and build on issues as they emerged [44].

Two local social science graduates – including MK – fluent in Chichewa, the language commonly spoken in Blantyre and one of two official national languages, collected the IDI and FGD data. That we did not encounter refusals in this study may be because households reporting chronic coughers had, at the enumeration phase, been informed about possible follow-up visits by a qualitative research team. Similarly, TB patients, who were all on treatment, might simply have welcomed the opportunity to engage with a health-related study. (We must mention that, through the informed consent process, data collectors clarified their role as researchers and not medical personnel, and also mentioned they had no influence on the healthcare services patients were receiving or might require in future.) Difficulties were also experienced in tracing TB patients owing to insufficient locator information in the TB register, or absent or invalid mobile phone details.

### Analysis and ethical issues

Full approval of the study was granted by the Malawi College of Medicine and the Human Sciences Research Council research ethics committees, and clearance to enter the communities requested from and provided by executive leaders of Blantyre and local leaders. Permission from the District Medical Officer and facility managers to access patients’ records was sought and granted. Health worker FGDs were scheduled to avoid causing disruption to services. Written informed consent and permission to record data were provided by all participants, with anonymity and confidentiality maintained at all stages. Participants were provided refreshments and reimbursed their transport costs.

FGDs and IDIs were recorded, transcribed and translated by trained personnel, checked for accuracy by (MK), and for intelligibility by (JC) who is fluent in Shona, a language related to Chichewa. Data were formally analysed using an inductive approach aligned to grounded theory. The transcripts were entered into NVivo data analysis software (Version 8, QSR International Pty Ltd, 2008) and coded based partly on questions brought into the study, and partly and to a large extent, inductively through identifying emerging concepts during analysis. In all instances and at all levels, coding entailed constant comparison [44]. Codes that belonged together were then grouped into families, and corresponding text retrieved under the codes. The families of codes were renamed according to the dominant issues, then split according to the families’ salient dimensions, and reconnected at more theoretical levels to generate broad categories or themes. The process was repeated until no new concepts or ways of connecting or breaking down data could be identified. JC performed the primary analysis, and MK, ELC and GH provided alternative perspectives. In addition to the triangulation of methods and data sources, therefore, the multidisciplinary research team’s analytical triangulation enhanced the truthfulness and credibility of the findings.

This paper focuses on the notion of control, one of the salient themes that emerged from the analysis. Other themes that emerged include the expectation on men to be providers (which is addressed in passing in this paper); the link between TB, HIV and healthcare-seeking; and care-seeking barriers at community and health service delivery levels. These themes will be detailed separately.

## Results

We focus on the notion of control’s form and significance to masculinity and, hence, healthcare-seeking behaviour. In both its form and emphasis, control emerged in ways not entirely foreseen at the study’s inception. It permeated the ways men handled an expectation to provide when employment opportunities are limited and incomes are small. Men’s sense of adequacy as men was thus perennially threatened, driving them to constantly worry about different sites where their emasculation might occur, including within their own families and the community.

### The expectation to be a competent provider

A division of responsibility by gender, whereby men work from outside the home and women are home-makers, was universally upheld.

*“… like most things … we should just assume* [laughs]*, well, what I wish him to be doing in the family, I mean, being employed, right?… because then it means that what I expect of him can be done. .. I can also say, ‘my husband works; end of the month he earns … and we can budget for this and that…’ because money is there.”* (IDI, 29-year old married mother of three)

It was considered critical that men have good foresight and anticipation, making failure to provide understandable only if arising from unavoidable or unexpected circumstances.

*“You have to be conscious of your responsibility … that every day you wake up, there is food.”* (Community men’s FGD)

*“I establish what is needed for the new day before going out, so I can bring it on return. Where things get hard, it has to be occasionally; then I admit that, although the wish was there, unavoidable impediments arose. Anything else I break down and procure just like that. Whenever I come home, children must scream, ‘Dad’s home!’”.* (30-year TB patient, father of two)

In addition, although day-to-day household matters were considered the wife’s responsibility, husbands were expected to lay down principles to guide the women.

*“… Before something arises, you want to know in advance. Your wife should give you ample time; and you should set your****principles****, that she must notify you before****pay****day.”* (Community men’s FGD)

If a woman failed, this therefore also implicated her male partner. On their part, and consistent with the view that masculinity is accomplished within the social realm [54, 55], men feared projecting a picture of being inept or lacking influence around their homes.

*“(she must) say at least a week before so I as the man don’t look like a fool. Because women contribute to making one look foolish by, for example, just saying from nowhere, ‘Maize flour is finished.’”* (Community men’s FGD)

Having wealth or a family whose entire needs were being met was seen as a key marker of masculinity. Importantly, men were also assessed and graded on the basis of what was termed “record”; for example, dressing in ways deemed appropriate, being articulate and commanding a presence when in the public domain, and having visibly healthy and happy children.

*“… a perfect man is independent, not a disgrace; someone dependable to his family, who fulfils their necessities so they don’t lack things; his wife must not move in torn clothes, or even lack maize flour.”* (Community men’s FGD)

*“In Malawian culture, he is perfect when he owns a family; is married. Someone is complete when he has children … every necessity … they say: a man should smell****moustache****.* [All laugh]*. Such is a real man, he has everything… toilet, granary … livestock … everything”* (Community men’s FGD)

*“When disagreements occur, parties must rely on you as someone to turn to* [mediate]*. So you must be able to speak… like a man.”* (30-year old father of two, TB patient)

Kind-heartedness, religiousness, and chastity mostly to protect one’s family from HIV/AIDS, were also widely espoused. However, these qualities seemed to bear more value when accompanying rather than displacing dominant and traditional attributes.

### Grappling with threats to masculinity and control

#### Financial threats

Where masculinities are organised into hierarchies, it is also the case that “most men are not actually in dominant positions, most of the time” [56]:29. The men in this study, living in cash-dependent urban settings where unemployment and low wages and incomes also prevailed, were portrayed as vulnerable, stretched, and constantly plagued by the threat of failure. The picture of unblemished masculinity depicted above, and clearly so much aspired to, seemed far removed from descriptions given of men’s actual lives.

*“Most men don’t measure up, because nowadays employment is scarce. In****town****[urban areas] we rely on money. If you can’t secure it, then you can’t support at all in the home. Most people are suffering.”* (Woman in community mixed sex FGD)

Men indicated they yearned for and derived gratification from success in their expected roles. A single father to three non-resident children lived with two brothers, a sister-in-law, and a niece in a house left by a deceased uncle. As they were not formally employed, the man and his brothers drew income from selling tap water from their home. The man, himself a TB patient, described his feelings regarding the support that he gave to his children.

*“They often come when they have no money. I try to find some to give them. It is things like those that leave joy in my heart; when my children come to me, and I accomplish what they want.”*

Yet, shouldering insurmountable responsibilities and battling to generate resources, sometimes exacerbated by disabling illness, men’s experiences were dominated more by pain and anxiety than by joy and gratification.

*“My wife, kids … grandmother at the village… other relatives … I do everything for them…. So when not working or doing any business, you’re a very poor person… Your big responsibilities become a big burden when you don’t send money. I spend months without sending to the village … and it’s painful.”* (33-year old father of two; chronic cougher)

*“… failing to do some things on my own… Like hard jobs, jobs that make me get money … I fail just because I have TB… It hinders me… I should do hard work but I have difficulties breathing. … When I am working and I start having difficulty breathing, I just leave it in the middle.”* (29-year old father of three; TB patient)

A 24-year old male TB patient who had just quit his job after relatives advised him to rest in order to recover described the mental strain from failing to support his parent as previously. Within his household where he stayed with his two brothers and a sister-in-law and her child, the man described feeling isolated because he was unable to contribute financially.

*“My mother’s condition at the village … sometimes she asks for money for fertilizer… But as I am now, the money is hard to get…. So I get depressed…* [Chuckles] *Also when you stay with someone … and you can’t contribute money…. to tell them you’re hungry… you can’t. So, whenever I get my hands on some money, I leave home and go out to eat somewhere.”*

#### Other sources of the threat of being considered ‘less than men’

Throughout, the accounts portray men grappling with tensions that revolve around their socially constructed images. Health problems, adverse economic conditions, and inability to achieve an omnipresence that permits monitoring all aspects of their lives and social contexts, all seemed to severely hinder men from achieving their desired representations.

The relational dynamics that accompany women’s involvement in income generation illustrate, in particular, men’s fears of losing control. Several women described heading and supporting their families when widowed, divorced, separated, or not living together with a spouse for work-related reasons. Some, however, took the role up even in their husbands’ presence, something both men and women acknowledged to be necessary in today’s economy.

Men then struggled to cope with the switching roles. Women bringing income or consolidating their hold over households for any other reason upset the gender role differentiations men were accustomed to and preferred. They threatened, in particular, men’s headship of and grasp over households, leading men to respond by simply disengaging further from the domestic space.

*“… men, regarding household work, a-ah, they don’t do* [laughing] *… They just stay at the****market****, huh! Like that, only coming home to sleep. Yeah* [laughing]*. That’s how they do. But … nowadays, there’s****gender****[equality], right? People assist each other. But men stick to that old life”* [laughing] (26-year married mother-of-two, who was running a grocery outlet)

Another concern for men was how they might naively believe they had greater say within households when, in fact, women quietly exercised such control.

*“We used to say the head of family is the man, yes… but that was only in the past. Nowadays some of us can get big-headed when, in fact, it is the woman who is wise… even wiser and cleverer in doing things than the man.”* (Community men’s FGD)

Despite their active efforts to dissociate themselves from feminine and domestic spaces, and increasing uncertainty about their role and influence within the home, men were conscious of the overwhelming workload burden on women. They were, however, ambivalent about helping out as they feared they would be considered effeminate.

*“You say ‘let me help out here’, and poof! goes respect for you. Afterwards, she relates to friends* [Laughter] *‘Me I am sorted, my children’s father, anything I leave out of place, he sorts nicely.’”* [Laughing] (Man in community mixed sex FGD)

*“Oh yes… when with friends, women talk carelessly, ‘don’t worry, let’s take our time talking, when I get home things are already done.’”* [Laughter] (Man in community mixed sex FGD)

Men suspected that, within their circles, women conspired to cast men in a poor light, influencing each other into extramarital sex and gossiping about husbands’ inadequacies, thus subjecting them to public scrutiny and ranking.

*“Speaking badly when chatting with their friends… Quarrels arise****daily****because of this; there are fights; homes are now like courts because women’s way of talking is not good. We feel disrespected”* (Man in community mixed sex FGD)

*“She mustn’t take domestic issues out to neighbours and friends, like how little her husband earns. If her neighbour’s husband makes a lot, this causes her to develop other - - - start prostitution.”* (Man in community mixed sex FGD)

*“They tell her, ‘Why suffer with an unemployed husband … End the marriage. Another man can marry and make you happy’ ”* (Man in community mixed sex FGD)

Furthermore, in men’s view, women used cunning skills to outwit their spouses, subtly and gradually eroding the latter’s influence and authority while covertly engaging in extramarital relationships.

*“You marry a woman and give her your****principles****. She breaks them****one-by-one****. She’ll tell you, ‘Your bad friend asked me out’, ‘What! I’ll deal with him’. Yet she is the****crook****, just blaming that other person because perhaps he caught her, and she must smear his character.”* (Community men’s FGD)

Women confirmed men’s concerns as they admitted using their networks or the space when husbands are at work to enter other sexual relationships.

*“We meet regularly and update: ‘How are things?’ If foolish you let all out … Just say you’re doing well. Then she fails to trap you … into being unfaithful”* (Woman in community mixed sex FGD)

*“Since we don’t go to work, we say: ‘He leaves in the morning, and returns in the evening. I sneak out, he won’t find out, or notice. By****three, four****, I’ll be back in’. It just takes two or three of similar minds.”* (Woman in community mixed sex FGD)*.*

Consequently, while they desired women’s involvement in cash generation, men seemed under pressure to strengthen control and vigilance lest the women engaged in extramarital sexual affairs or became domineering and defiant in the home.

*“The thinking is, a man gets money through honest means, but a woman must give a detailed explanation.”* (Man in community mixed sex FGD)

*“He can leave home, go do piece-work, and bring money. For the woman to do the same, and say ‘I did a small job somewhere’, no one will buy that …”* (Woman in community mixed sex FGD)

*“Now the woman brings problems from outside into the house, and when he questions, she reminds him ‘I feed you, and pay the rent!’”* (Man in community mixed sex FGD)

### Actions to deal with threats to masculinity

As noted earlier, men partly deal with threats to their preferred images by dissociating from what is domestic and feminine. In some instances, this involves amplifying the extent to which they conform to signifiers of dominant masculinity such as resilience and control. One participant drew attention to how, in spite of his slight stature and youthful age, he still managed to achieve big things (“It’s something pleasing with this age that I am; I look short but I am big”). Demonstrating how he stayed above the situation, the man boasted about easily controlling his wife’s movements and also being a reliable provider which kept his wife steadfastly faithful, unlike other marriages.

*“I can tell her to stop going to church; and she will. Also, she works. I told her she must stop. She loves marriage so she quit. … A woman follows (bad advice) because of the state of the man’s pocket … If you’re poor, she goes to others … even telling you it’s because you’ve nothing. But me, I feel she counts on me a lot.”* (28-year old married father of two; chronic cougher)

Frequently mentioned as a reason why women have extramarital sexual relationships, adequate provision was thus seen by men as essential for avoiding destabilisation of their family by unscrupulous members of the community. Violence – a key marker of manhood [57] – was also sometimes contemplated. Moreover, re-affirming how ‘self-reliance’ [58] also defines masculinity, men saw ‘singlehanded’ resolution of problems as critical.

*“I want to look after everything in the home so it’s not disgraceful. … I try by all means to deal with problems on my own. .. But when you do well in your home … some people try to disturb, even whilst you’re there … trying violent tactics to disturb the lifestyle you are leading… when people come like for violence, avoid a bit so they can change.”* (30-year old father of two; TB patient)

The accounts of men continued to accentuate masculinity where, in their view, it was increasingly threatened when women earned income. Men adamantly stressed that they were and needed to be recognised as heads of homes irrespective of the income they or their wives brought.

*“A man being the head takes the lead in all of his family’s activities, whether working or not; or whether it’s the wife who is working…”* (Community men’s FGD)

*“Earning doesn’t prove she is ahead, no. The man is the head. … That man must take charge of the family property, it does not matter if the woman brings in a lot … she must support the man in his point of view.”* (Community men’s FGD)

Troubled by what to them was unmanageable disruption that followed when a wife earned, men went as far as turning to their marriage vows just to emphasize that women must always be deferent.

*“There has to be mutual understanding between the woman and her husband … When you form a family, you promise to be one body until death… So men expect the woman to be submissive, so as to be together … money or no money”* (Community men’s FGD)

### Masculinity representations and illness response

A key goal of this study was to understand how masculinity affects healthcare-seeking. While the previous sections outlined the form and manifestations of the notion of control, this section elaborates on the impact on men’s expected responses when ill. The section also presents perspectives that emerged regarding masculine strength, and how it also influences the health responses.

#### Men’s expected response when ill

Although it was felt that men now needed to promptly seek healthcare mainly because of the HIV/AIDS epidemic, a widespread view among both men and women was that men should wait until symptoms became unbearable, or use exercise to rid the body of disease. Acknowledging symptoms or seeking care too early was considered bad for minor illnesses.

*“I’m supposed to be active, chasing it out. We do a bit of****exercise****and those small diseases vanish.”* (26-year old unmarried male TB patient)

*“You only seek help when you see that you’re really sick, realising: ‘Oh, I can’t even walk; a few steps and I must sit down.’ Meaning you can’t work either. Only then am I supposed to consider it.*” (31-year old father of three, TB patient)

Being household heads was said to require that men publicly display stoicism even while suffering in private. By showing strength and resilience in the face of challenges, in ways that even drew awe from onlookers, men virtually defended their status in the home while, at the same time, assuming a protective and re-assuring stance to their dependants. A 31-year old father of three and TB patient explained:

*“If you have to consider pain, just remember then that … those who come to you [depend on you for help] will as well know they’re just going to starve … You want to be able to tell people: ‘I went to such-such a place even with my body not well’ and they’ll be shocked”*

As much as they tried, however, men acknowledged it was not possible to ignore some of the more serious illnesses.

*“Sickness as this one that’s struck me, the one causing me to cough a lot… no matter how hard one tries to avoid it, one just has to lie down.”* (IDI, 30-year man, TB patient)

Interestingly, a global look into the accounts indicates that personal experience of TB illness, diagnosis and treatment may have changed our male participants’ views regarding the wisdom of delaying seeking care. Generally, male TB patients would repeatedly express stereotypically masculine views, describing how they had delayed healthcare seeking out of stoicism, or used alcohol to manage pain or ‘expel’ disease. But then, in a clear shift in perspective as exemplified by the preceding quote, these participants would also endorse early healthcare seeking.

#### Conceptions of bodily strength in men

Discussions of masculinity and healthcare-seeking often highlight men’s self-perception as being strong, or stoicism. Although the accounts confirmed these views to an extent, the relationship between manhood, the body, and strength emerged in complex ways.

To start with, sexual competence was exalted and linked to bodily strength. Of significance, women also underscored this concept. This reinforced it as a relational construct [23] that put even more pressure on men to repudiate weakness and femininity [57].

*“When we say ‘this is a man’, it’s because he’s strong, right? … We expect that when he decides to marry, he has the strength. We all know about this strength, right? … ‘I’m grown up and I can do this.’ But when having sexual intercourse … some just lie; they do nothing, proving he doesn’t have strength”* (Community women’s FGD)

Regarding vulnerability to illness and sensitivity to bodily pain, however, participants struggled to pin down the differences between men and women, and instead shifted and contradicted positions. In isolated instances participants of both sexes, but more often women, pronounced that men’s bodies were not any stronger than women’s. (“They have flesh, so men should also feel pain just the same way women do.” Community women’s FGD). Men were seen, rather, as being under pressure to remain healthy or, that failing, uphold a facade of being strong.

*“Often they hide … they don’t want to tell you they’re sick. You just suffer inside, ‘My husband is sick… clearly with something serious…’ But he hides; tries to be strong.”* (Woman in community mixed sex FGD)

Men themselves caricatured their display of stoicism while suffering privately. They acknowledged how their delay in recognising or admitting being ill meant they not only entered care in worse state than women, but also proceeded to fare worse.

*“You can be suffering but still be up and walking. They could tell you at home, ‘You don’t look well’, but you’ll keep walking [moving around]”.* [Animated laughter and discussion] *(*Man in community mixed sex FGD)

*“Look closely at the****hospitals****, most men in there are worse [than women], because they take time to acknowledge, saying instead ‘Me, I’m not suffering from this*’” (30-year old father of two; TB patient)

The perception that men are ultimately stronger and able to withstand disease nevertheless remained prominent. Men’s strength was attributed to their natural build and inclination to perform strenuous tasks.

*“We can say they were given [created with] strong bones; whereas women, we were given weaker bones.”* (46-yr old widow; TB patient)

*“Wherever he goes, it’s never by bus. He runs about, doing many things. This****exercise****makes the body strong and able to withstand disease unlike women …”* (Man in community mixed sex FGD)

*“The activities we often do … I’ve seen women who, because they do manly tasks, are very strong… when sick, they differ from the other women”* (55-year old father of five; TB patient)

Paradoxically, the strong bodies derived from men’s constant exercise are said to become more vulnerable when serious disease does attack. Looking closely, the participants could be referring to deleterious effects of serious disease ignored by men for too long.

*“Because a man works hard, whenever attacked by disease, the disease’s strength and that of the body, when these two meet, he goes****flat****.”* (Woman in community mixed sex FGD)

In rare instances, women were described as stronger because they continued with their daily chores while sick, whereas men had the ‘luxury’ to stay in bed. This too may refer to advanced illness stages when men, earlier said to be stoical, become bedridden with late-stage, previously ignored illness.

*“Say a woman coughs; you don’t find her in bed. She’ll still do her chores. But men will sleep [lie down] from morning* [laughter] *till sunset… to the point of having food follow them* [laughter] *to the bedroom.”* (Women’s community FGD)…

## Discussion

Two factors primarily motivated this study: the resurgence of TB as a major public health problem on the African continent, and men’s well documented delay in seeking care, generally and specifically for TB and HIV/AIDs. With limited prior understandings of the topic, an exploratory approach was used, where chronic cough – a known TB symptom and condition more likely to be witnessed and recognised at the community and lay levels than TB – was the main entry point for the investigation. Tuberculosis patients and specific questions on TB were still included to maintain the study’s primary focus on TB. The findings therefore illuminate determinants of men’s healthcare seeking more generally, yet in ways still relevant for TB.

The paper raises two significant and closely related issues. Firstly, men in this poor urban community experience immense pressure to live up to masculine ideals that their environment and circumstances, and sometimes health status, simply do not support. Secondly, although important in shaping men’s perspectives and health behaviours, some notions of masculinity are not consistently held or embraced by either men or women; this has implications for the notions’ application in work with men.

Consistent with the literature [20, 59], control emerged as a concept that underlines masculinity in this population. Key and valued aspects of masculinity included managing one’s household with grip and foresight, providing adequately for the family, monitoring one’s public image, and controlling wives’ movements and sexuality. The expectations, however, diverged widely from the reality of constraints faced by men living in challenging economic circumstances, especially when also dealing with poor health. Struggling to raise income, men risked being publicly devalued while their wives might look for other sexual partners. Men wished for but were unable to achieve the omnipresence required to monitor their wives or the way the men themselves are perceived by the public. The crisis they experience as a result is articulated in their descriptions of failing to measure up; their worries trying to protect or improve their personas as ‘adequate men’; their pleas to wives not to use income leverage to offset gender power hierarchies in homes; and the fears around having token leadership in their homes.

Differentiation, grading, and exclusion of other men and women are key to masculine identity [59, 60]. To avert being seen as feminine or as occupiers of feminine space, men embark on a ‘flight from the feminine … repudiation of femininity” [57]:122. In other words, the challenges men face in living up to desired masculinity images only lead to intensified effort [57], or amplification of those versions that are available even if ranked lower – a form of “compensatory” behaviour [54].

How might one explain the scenario of fracture, paranoia and suspicion and how it has come to occupy men’s psyches, social lives and identities? As an explanatory framework, social constructionism [61] seems apt given the multiple and multi-level determinants of gender, its shifts across time and context, and the well documented contradictions in masculinities. Moreover, most masculinity literature has come from the global North. Although some of the concepts apply cross-regionally, others are specific to certain contexts and their histories [22, 62]. We examine below the role of historical and contemporary structural dynamics in shaping masculinities as expressed in Southern African settings today.

In some settings in Southern Africa, the binary allocation of women and men respectively into domestic and public spaces, and provider and dependant roles have been linked to Christian and colonial interventions that ignored or deliberately altered existing gender relations [63–65]. Women’s access and rights to land were curtailed [66, 67] as policies were enacted to facilitate access to cheap male labour for mines and farms [68]. The declining land productivity in villages, growing reliance on cash crops, and increased workloads in the absence of men, coupled with absent or indeterminate title to land for women, resulted in women becoming increasingly poorer and over-exerted [66]. The rural, domestic, feminine, and non-monetised became devalued and synonymous with poverty. This explains in part the manifestation of masculinity today, where emphasis is put, as in this study, on maintaining distance from femininity and domestic spaces. Added to the historical factors, continuing socio-economic and political changes, including globalisation, have fanned growing consumerism amidst inequality and poverty [69]. Work intermittence has similarly risen as a permanent condition [70], while women are increasingly taking part in the labour market [71]. These developments have further undermined the position of men and exacerbated the masculinity crisis.

Literature identifies some attributes as key to defining masculinity [54, 58], implying a measure of stability and continuity around them. In the current study, the lack of consistency around the concept of men’s bodily strength or the responses deemed appropriate for men in illness is therefore striking. The masculinity literature nevertheless acknowledges plurality and flux existing alongside continuity and stability as partly responsible for the current masculinity crisis. In one sense, our use of the more flexible qualitative approach helped draw out complexities as participants formed, explored and shifted opinions [72, 73]. For TB patients specifically, shifting or contradictory perspectives might indicate the effects of the experience of illness, disease diagnosis, and treatment on their insights.

Alternatively, the accounts illuminate the internal conflicts related to performing for public consumption [74] masculinities that are also clearly harmful. Because masculinities are multiple, competitive, hierarchical and contradictory [21, 57, 58, 75], and needing constant reaffirmation in the face of multiple threats, some behaviours are then deployed to elicit feedback and draw awe. It is inevitable that some practices also become noticeable as sources of harm (as in the case of the deliberate delay to seek healthcare until *in extremis*) and, increasingly, individuals learn about or contemplate alternative and less harmful ways to be masculine. The FGDs and IDI conversations would, in this instance, provide men with a rare space and opportunity to confront, explore, and evaluate in a sustained and focused manner their perspectives and behaviours. In turn, this led to the embrace of alternative masculinity versions and the querying of conventional ones, pointing to what Inhorn [76] terms “emergent masculinities”, or “ongoing, context specific, embodied changes within men’s enactments of masculinity” (p 802). Having said this, Inhorn’s use of “emergent masculinities” draws from long-term ethnographic fieldwork, whereas we adopt the notion here in the context of a grounded theory analysis based on cross-sectional qualitative data.

## Conclusions

Facilitating men to seek care early is an urgent public health imperative, in view of the contexts of high HIV/AIDS prevalence but increasingly available treatment, and the role of care-seeking delay in maintaining TB transmission. Within the nascent field of masculinity and health studies in Africa, emerging research has thus begun to focus on HIV/AIDS [77, 78]. The way masculinity emerged in this study indicates the importance of continuing to build on the growing body of work on masculinity and health in African settings, and specifically to complement survey-type methods with flexible designs capable of illuminating complexity and providing critical information to inform interventions. From this study, control seems particularly central in men’s lives and to their engagement with their health. The complex manifestations of masculinity reported here suggest the need for interventions targeting men in health and TB control to assume supportive, multidimensional and long-term outlooks. There is also need to approach common assumptions about masculinity cautiously, while the signs of “emergent masculinities” can provide a useful platform from which to support the transformation of harmful masculinity. A limitation is that the findings pertain to a specific case, namely men and women living in specific communities under specific socio-economic circumstances. They are thus generalizable, in the first instance, to intrinsically similar communities. However, comparable findings from studies elsewhere in the region [25, 34, 78] imply there is possibility to generalise the findings more widely.
